# Bis[benzyl 3-(3-phenyl­prop-2-enyl­idene)dithio­carbazato-κ^2^ *N* ^3^,*S*]cadmium

**DOI:** 10.1107/S1600536812028127

**Published:** 2012-06-30

**Authors:** M. S. Reza, M. A. A. A. A. Islam, M. T. H. Tarafder, M. C. Sheikh, E. Zangrando

**Affiliations:** a Department of Chemistry, Rajshahi University of Engineering and Technology, Rajshahi 6204, Bangladesh; b Department of Chemistry, Rajshahi University, Rajshahi 6205, Bangladesh; c Department of Applied Chemistry, Faculty of Engineering, University of Toyama, 3190 Gofuku, Toyama 930-8555, Japan; d Department of Chemical and Pharmaceutical Science, Via L. Giorgieri 1, 34127 Trieste, Italy

## Abstract

In the title complex, [Cd(C_17_H_15_N_2_S_2_)_2_], the Cd^II^ ion is located on a twofold rotation axis and exhibits a coordination number of four within a very distorted coordination environment that is best described as bis­phenoidal. The two deprotonated Schiff base ligands chelate the Cd^II^ ion through the azomethine N and the thiol­ate S atom. The dihedral angle between the two chelating ligands is 84.01 (9)°. Weak inter­molecular C—H⋯S inter­actions lead to the formation of chains along the *c* axis.

## Related literature

For the structure of uncoordinated Schiff bases, see: Tarafder, Crouse *et al.* (2008 ▶); Tarafder, Islam *et al.* (2008 ▶). For the isotypic Zn and Hg analogues, see: Fun *et al.* (2008 ▶); Islam *et al.* (2012 ▶). For the coordination behaviour of metal ions (Co, Ni, Cu, Zn, Cd and Hg) with the cinnamaldehyde Schiff base of *S*-methyl­dithio­carbazate, see: Liu *et al.* (2009 ▶); Abram *et al.* (2006 ▶). For the bioactivity of transition metal complexes of similar Schiff base ligands, see: Chew *et al.* (2004 ▶); How *et al.* (2008 ▶); Maia *et al.* (2010 ▶).
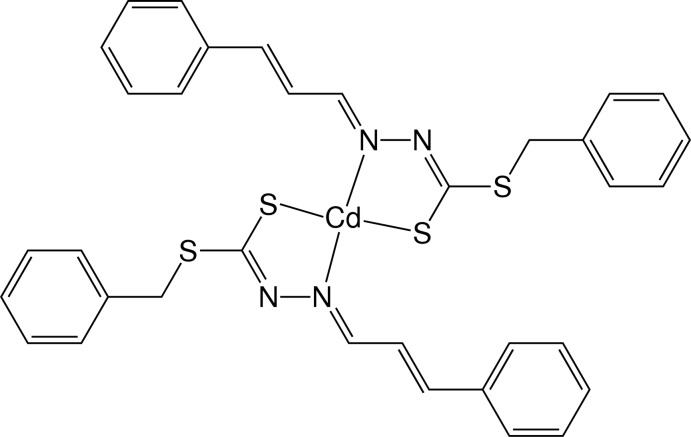


## Experimental

### 

#### Crystal data

[Cd(C_17_H_15_N_2_S_2_)_2_]
*M* *_r_* = 735.26
Orthorhombic, 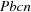

*a* = 36.2497 (7) Å
*b* = 9.9940 (2) Å
*c* = 8.9392 (2) Å
*V* = 3238.49 (12) Å^3^
*Z* = 4
Cu *K*α radiation
μ = 8.05 mm^−1^
*T* = 173 K
0.3 × 0.3 × 0.1 mm

#### Data collection

Rigaku R-AXIS RAPID diffractometer
Absorption correction: multi-scan (*ABSCOR*; Rigaku, 1995 ▶) *T*_min_ = 0.325, *T*_max_ = 0.448
10761 measured reflections
2964 independent reflections
2439 reflections with *I* > 2σ(*I*)
*R*_int_ = 0.079

#### Refinement

*R*[*F* ^2^ > 2σ(*F* ^2^)] = 0.042
*wR*(*F* ^2^) = 0.115
*S* = 1.02
2964 reflections
195 parameters
H-atom parameters constrained
Δρ_max_ = 1.36 e Å^−3^
Δρ_min_ = −0.52 e Å^−3^

### 

Data collection: *RAPID-AUTO* (Rigaku, 1995 ▶); cell refinement: *RAPID-AUTO*; data reduction: *RAPID-AUTO*; program(s) used to solve structure: *SHELXS97* (Sheldrick, 2008 ▶); program(s) used to refine structure: *SHELXL97* (Sheldrick, 2008 ▶); molecular graphics: *CrystalStructure* (Rigaku, 2010 ▶); software used to prepare material for publication: *publCIF* (Westrip, 2010 ▶).

## Supplementary Material

Crystal structure: contains datablock(s) I, global. DOI: 10.1107/S1600536812028127/wm2651sup1.cif

Structure factors: contains datablock(s) I. DOI: 10.1107/S1600536812028127/wm2651Isup2.hkl

Additional supplementary materials:  crystallographic information; 3D view; checkCIF report

## Figures and Tables

**Table:** 

Cd—N1	2.306 (2)
Cd—S1	2.4285 (9)

**Table:** 

N1^i^—Cd—N1	103.00 (12)
N1^i^—Cd—S1	119.99 (6)
N1—Cd—S1	80.27 (6)
S1—Cd—S1^i^	149.19 (5)

Symmetry code: (i) 
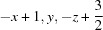
.

**Table 2:** Hydrogen-bond geometry (Å, °)

*D*—H⋯*A*	*D*—H	H⋯*A*	*D*⋯*A*	*D*—H⋯*A*
C13—H13⋯S2^ii^	0.95	2.75	3.690 (4)	170

Symmetry code: (ii) 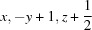.

**Fig. 1.**
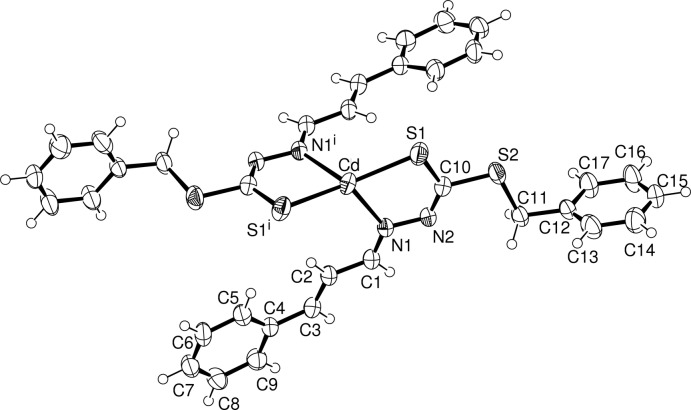
An ORTEP drawing (ellipsoids at the 50% probability level) of the title compound with the atom labelling scheme. [Symmetry code: (i) -x + 1, y, -z + 3/2.]

## supplementary crystallographic information

### Comment

In continuation of our interest in exploring the chemistry of Schiff bases derived from *S*-benzyldithiocarbazate (Tarafder, Crouse *et al.*, 2008; Tarafder, Islam *et al.*, 2008) and of their metal complexes, due to their intriguing coordination behaviour, physico-chemical properties, and potential biological activities, we have completed the syntheses of group 12 metal complexes with the same Schiff base, *viz.* bis[benzyl N'-(3-phenylprop-2-enylidene)dithiocarbazate]. For the coordination behaviour of metal ions (Co, Ni, Cu, Zn, Cd, and Hg) with the cinnamaldehyde Schiff base of *S*-methyldithiocarbazate, see: Liu *et al.* (2009); Abram *et al.*, (2006). For the bioactivity of transition metal complexes of similar Schiff base ligands, see: Chew *et al.* (2004); How *et al.* (2008); Maia *et al.* (2010).

In the present complex the Cd^II^ ion lies on a twofold rotation axis and therefore the asymmetric unit contains one-half of the molecule (Fig. 1). The fourfold coordination is best described as bisphenoidal, with the Cd^II^ ion being chelated by two benzyl *N*'-(3-phenylprop-2-enylidene)dithiocarbazate ligands through the azomethine nitrogen and the thiolate sulfur donors. The two chelating five-membered rings form a dihedral angle of 84.01 (9)°. Since the structure is isotypic with those of Zn (Fun *et al.*, 2008) and Hg (Islam *et al.*, 2012), it is worthwhile to compare the geometries around the metal ions. The *M*—N bond lengths in the series follow the trend Zn < Cd < Hg (2.0662 (12), 2.306 (2), 2.489 (3) Å) in agreement with the respective ionic radii. On the other hand, among the *M*—S bond lengths, the Cd—S one is the longest, with values of 2.2636 (4), 2.4285 (9), 2.3668 (11) Å along this series. Moreover, the bite angle N1—Cd—S1 of 80.27 (6)° is in between the values for the Zn (86.96 (3)°) and Hg (77.93 (6)°) complexes.

The crystal packing is consolidated by weak C13–H13···S2 interactions , giving rise to a chain motif extending along the *c* axis.

### Experimental

The Schiff base, benzyl *N*'-(3-phenylprop-2-enylidene)hydrazinecarbodithioate was prepared as prevoiusly reported (Tarafder, Islam *et al.*, 2008). Cadmium(II) acetate dihydrate (0.066 g, 0.25 mmol) dissolved in absolute ethanol (20 ml) was added to a hot absolute ethanol solution (50 ml) of the Schiff base (0.163 g, 0.5 mmol) under refluxing condition, which was continued for 2 h. The yellow precipitate which formed was filtered off, washed with hot ethanol and dried *in vacuo* over anhydrous CaCl_2_. Yield: 0.199 g (87%). 54 mg of the compound was dissolved in chloroform (10 ml) at room temperature and mixed with toluene (5 ml). The resultant solution was allowed to stand at ambient temperature. Yellow square-shaped flat single crystals developed after 7 days. (m.p.= 438 K).

### Refinement

All H atoms were geometrically located and treated as riding atoms, with C—H = 0.95 Å for C(aromatic) and 0.99 Å, for C(methylene), with *U*_iso_(H) = 1.2*U*_eq_(C). The highest residual electron density peak (1.36 e Å^-3^) is located at 1.09 Å from the Cd atom.

### Crystal data

**Table:**

[Cd(C_17_H_15_N_2_S_2_)_2_]	*F*(000) = 1496
*M**_r_* = 735.26	*D*_x_ = 1.508 Mg m^−^^3^
Orthorhombic, *P**b**c**n*	Cu *K*α radiation, λ = 1.54187 Å
Hall symbol: -P 2n 2ab	Cell parameters from 29242 reflections
*a* = 36.2497 (7) Å	θ = 3.7–68.2°
*b* = 9.9940 (2) Å	µ = 8.05 mm^−^^1^
*c* = 8.9392 (2) Å	*T* = 173 K
*V* = 3238.49 (12) Å^3^	Prism, yellow
*Z* = 4	0.3 × 0.3 × 0.1 mm

### Data collection

**Table:**

Rigaku R-AXIS RAPID diffractometer	2964 independent reflections
Radiation source: fine-focus sealed tube	2439 reflections with *I* > 2σ(*I*)
Graphite monochromator	*R*_int_ = 0.079
Detector resolution: 10.000 pixels mm^-1^	θ_max_ = 68.3°, θ_min_ = 4.6°
ω scans	*h* = −42→43
Absorption correction: multi-scan (*ABSCOR*; Rigaku, 1995)	*k* = −11→12
*T*_min_ = 0.325, *T*_max_ = 0.448	*l* = −10→10
10761 measured reflections	

### Refinement

**Table:**

Refinement on *F*^2^	Primary atom site location: structure-invariant direct methods
Least-squares matrix: full	Secondary atom site location: difference Fourier map
*R*[*F*^2^ > 2σ(*F*^2^)] = 0.042	Hydrogen site location: inferred from neighbouring sites
*wR*(*F*^2^) = 0.115	H-atom parameters constrained
*S* = 1.02	*w* = 1/[σ^2^(*F*_o_^2^) + (0.0606*P*)^2^] where *P* = (*F*_o_^2^ + 2*F*_c_^2^)/3
2964 reflections	(Δ/σ)_max_ = 0.001
195 parameters	Δρ_max_ = 1.36 e Å^−^^3^
0 restraints	Δρ_min_ = −0.52 e Å^−^^3^

### Special details

**Table:**

Geometry. All s.u.'s (except the s.u. in the dihedral angle between two l.s. planes) are estimated using the full covariance matrix. The cell s.u.'s are taken into account individually in the estimation of s.u.'s in distances, angles and torsion angles; correlations between s.u.'s in cell parameters are only used when they are defined by crystal symmetry. An approximate (isotropic) treatment of cell s.u.'s is used for estimating s.u.'s involving l.s. planes.
Refinement. Refinement of *F*^2^ against ALL reflections. The weighted *R*-factor *wR* and goodness of fit *S* are based on *F*^2^, conventional *R*-factors *R* are based on *F*, with *F* set to zero for negative *F*^2^. The threshold expression of *F*^2^ > 2σ(*F*^2^) is used only for calculating *R*-factors(gt) *etc*. and is not relevant to the choice of reflections for refinement. *R*-factors based on *F*^2^ are statistically about twice as large as those based on *F*, and *R*- factors based on ALL data will be even larger.

### Fractional atomic coordinates and isotropic or equivalent isotropic displacement parameters (Å^2^)

**Table:**

	*x*	*y*	*z*	*U*_iso_*/*U*_eq_	
Cd	0.5000	0.44301 (4)	0.7500	0.04150 (16)	
N1	0.52779 (6)	0.2994 (3)	0.9175 (3)	0.0358 (6)	
N2	0.56616 (6)	0.2968 (3)	0.9179 (3)	0.0367 (6)	
S1	0.56402 (2)	0.50756 (11)	0.71527 (10)	0.0483 (3)	
S2	0.62987 (2)	0.39583 (11)	0.83639 (11)	0.0540 (3)	
C1	0.51250 (9)	0.2128 (3)	1.0048 (3)	0.0398 (7)	
H1	0.5276	0.1511	1.0578	0.048*	
C2	0.47320 (8)	0.2078 (3)	1.0235 (3)	0.0395 (7)	
H2	0.4587	0.2715	0.9710	0.047*	
C3	0.45573 (8)	0.1191 (4)	1.1103 (4)	0.0415 (8)	
H3	0.4705	0.0526	1.1570	0.050*	
C4	0.41607 (8)	0.1147 (3)	1.1403 (3)	0.0381 (7)	
C5	0.39164 (8)	0.2100 (4)	1.0849 (4)	0.0436 (8)	
H5	0.4007	0.2808	1.0244	0.052*	
C6	0.35458 (8)	0.2033 (4)	1.1162 (4)	0.0476 (9)	
H6	0.3383	0.2695	1.0779	0.057*	
C7	0.34086 (9)	0.1000 (4)	1.2038 (4)	0.0511 (9)	
H7	0.3152	0.0949	1.2243	0.061*	
C8	0.36437 (11)	0.0055 (5)	1.2607 (4)	0.0516 (10)	
H8	0.3550	−0.0643	1.3220	0.062*	
C9	0.40186 (10)	0.0116 (5)	1.2288 (4)	0.0455 (8)	
H9	0.4180	−0.0550	1.2675	0.055*	
C10	0.58178 (8)	0.3856 (3)	0.8347 (3)	0.0391 (7)	
C11	0.64403 (8)	0.2893 (4)	0.9912 (4)	0.0455 (8)	
H11A	0.6300	0.3118	1.0829	0.055*	
H11B	0.6399	0.1939	0.9668	0.055*	
C12	0.68464 (8)	0.3169 (3)	1.0126 (3)	0.0413 (8)	
C13	0.69639 (9)	0.4203 (4)	1.1010 (5)	0.0577 (10)	
H13	0.6787	0.4747	1.1506	0.069*	
C14	0.73377 (10)	0.4472 (4)	1.1193 (5)	0.0619 (12)	
H14	0.7415	0.5188	1.1818	0.074*	
C15	0.75932 (9)	0.3706 (4)	1.0474 (4)	0.0538 (10)	
H15	0.7849	0.3884	1.0598	0.065*	
C16	0.74806 (8)	0.2689 (4)	0.9579 (5)	0.0593 (10)	
H16	0.7659	0.2167	0.9065	0.071*	
C17	0.71095 (8)	0.2401 (4)	0.9404 (4)	0.0517 (9)	
H17	0.7035	0.1675	0.8787	0.062*	

### Atomic displacement parameters (Å^2^)

**Table:**

	*U*^11^	*U*^22^	*U*^33^	*U*^12^	*U*^13^	*U*^23^
Cd	0.0334 (2)	0.0470 (3)	0.0440 (2)	0.000	−0.01208 (12)	0.000
N1	0.0262 (12)	0.0447 (16)	0.0365 (13)	0.0008 (11)	−0.0011 (10)	0.0027 (12)
N2	0.0255 (12)	0.0478 (17)	0.0368 (13)	0.0013 (11)	0.0000 (10)	0.0006 (12)
S1	0.0401 (5)	0.0524 (6)	0.0525 (5)	−0.0107 (4)	−0.0125 (4)	0.0125 (4)
S2	0.0278 (4)	0.0780 (7)	0.0562 (5)	−0.0073 (4)	0.0006 (3)	0.0214 (5)
C1	0.0286 (14)	0.050 (2)	0.0411 (16)	0.0016 (14)	−0.0024 (13)	0.0023 (15)
C2	0.0269 (14)	0.051 (2)	0.0408 (16)	0.0021 (14)	−0.0023 (12)	0.0052 (15)
C3	0.0308 (16)	0.052 (2)	0.0422 (17)	0.0037 (14)	−0.0054 (13)	0.0017 (15)
C4	0.0285 (15)	0.049 (2)	0.0368 (16)	−0.0030 (13)	−0.0014 (12)	−0.0023 (14)
C5	0.0337 (16)	0.049 (2)	0.0481 (18)	−0.0035 (14)	0.0000 (14)	0.0018 (16)
C6	0.0289 (16)	0.056 (2)	0.058 (2)	−0.0016 (15)	−0.0051 (14)	−0.0033 (18)
C7	0.0328 (18)	0.068 (3)	0.053 (2)	−0.0074 (17)	0.0038 (16)	−0.013 (2)
C8	0.044 (2)	0.058 (3)	0.054 (2)	−0.013 (2)	0.0063 (14)	0.0020 (17)
C9	0.0410 (19)	0.047 (2)	0.0486 (19)	0.0003 (17)	−0.0010 (14)	0.0020 (16)
C10	0.0301 (15)	0.049 (2)	0.0382 (16)	−0.0005 (14)	−0.0046 (12)	0.0002 (15)
C11	0.0256 (15)	0.060 (2)	0.0504 (19)	0.0024 (14)	0.0032 (13)	0.0102 (17)
C12	0.0256 (15)	0.055 (2)	0.0438 (17)	0.0007 (13)	0.0037 (12)	0.0050 (16)
C13	0.0376 (19)	0.071 (3)	0.065 (3)	0.0062 (17)	0.0113 (17)	−0.014 (2)
C14	0.046 (2)	0.075 (3)	0.065 (3)	−0.0093 (19)	−0.0016 (18)	−0.017 (2)
C15	0.0273 (16)	0.077 (3)	0.057 (2)	−0.0049 (17)	−0.0021 (15)	0.004 (2)
C16	0.0286 (18)	0.068 (3)	0.081 (3)	0.0077 (17)	0.0040 (16)	−0.010 (2)
C17	0.0330 (17)	0.054 (2)	0.068 (2)	0.0011 (15)	0.0028 (16)	−0.0126 (19)

### Geometric parameters (Å, º)

**Table:**

Cd—N1^i^	2.306 (2)	C6—H6	0.9500
Cd—N1	2.306 (2)	C7—C8	1.370 (6)
Cd—S1	2.4285 (9)	C7—H7	0.9500
Cd—S1^i^	2.4285 (9)	C8—C9	1.390 (5)
N1—C1	1.291 (4)	C8—H8	0.9500
N1—N2	1.391 (3)	C9—H9	0.9500
N2—C10	1.288 (4)	C11—C12	1.510 (4)
S1—C10	1.744 (3)	C11—H11A	0.9900
S2—C10	1.746 (3)	C11—H11B	0.9900
S2—C11	1.819 (3)	C12—C13	1.368 (5)
C1—C2	1.435 (4)	C12—C17	1.384 (4)
C1—H1	0.9500	C13—C14	1.391 (4)
C2—C3	1.337 (4)	C13—H13	0.9500
C2—H2	0.9500	C14—C15	1.362 (5)
C3—C4	1.463 (4)	C14—H14	0.9500
C3—H3	0.9500	C15—C16	1.356 (5)
C4—C5	1.392 (4)	C15—H15	0.9500
C4—C9	1.398 (5)	C16—C17	1.385 (4)
C5—C6	1.374 (4)	C16—H16	0.9500
C5—H5	0.9500	C17—H17	0.9500
C6—C7	1.388 (5)		
			
N1^i^—Cd—N1	103.00 (12)	C7—C8—C9	120.2 (4)
N1^i^—Cd—S1	119.99 (6)	C7—C8—H8	119.9
N1—Cd—S1	80.27 (6)	C9—C8—H8	119.9
N1^i^—Cd—S1^i^	80.27 (6)	C8—C9—C4	120.6 (4)
N1—Cd—S1^i^	119.99 (6)	C8—C9—H9	119.7
S1—Cd—S1^i^	149.19 (5)	C4—C9—H9	119.7
C1—N1—N2	114.6 (2)	N2—C10—S1	132.2 (2)
C1—N1—Cd	128.5 (2)	N2—C10—S2	118.3 (2)
N2—N1—Cd	116.73 (17)	S1—C10—S2	109.45 (17)
C10—N2—N1	115.2 (2)	C12—C11—S2	105.4 (2)
C10—S1—Cd	95.10 (11)	C12—C11—H11A	110.7
C10—S2—C11	104.71 (14)	S2—C11—H11A	110.7
N1—C1—C2	121.3 (3)	C12—C11—H11B	110.7
N1—C1—H1	119.3	S2—C11—H11B	110.7
C2—C1—H1	119.3	H11A—C11—H11B	108.8
C3—C2—C1	124.1 (3)	C13—C12—C17	118.2 (3)
C3—C2—H2	117.9	C13—C12—C11	121.0 (3)
C1—C2—H2	117.9	C17—C12—C11	120.8 (3)
C2—C3—C4	126.3 (3)	C12—C13—C14	121.1 (3)
C2—C3—H3	116.9	C12—C13—H13	119.4
C4—C3—H3	116.9	C14—C13—H13	119.4
C5—C4—C9	118.1 (3)	C15—C14—C13	119.9 (4)
C5—C4—C3	122.7 (3)	C15—C14—H14	120.1
C9—C4—C3	119.2 (3)	C13—C14—H14	120.1
C6—C5—C4	121.1 (3)	C16—C15—C14	119.6 (3)
C6—C5—H5	119.5	C16—C15—H15	120.2
C4—C5—H5	119.5	C14—C15—H15	120.2
C5—C6—C7	120.1 (3)	C15—C16—C17	121.0 (3)
C5—C6—H6	119.9	C15—C16—H16	119.5
C7—C6—H6	119.9	C17—C16—H16	119.5
C8—C7—C6	119.9 (3)	C12—C17—C16	120.1 (3)
C8—C7—H7	120.0	C12—C17—H17	119.9
C6—C7—H7	120.0	C16—C17—H17	119.9
			
N1^i^—Cd—N1—C1	−62.5 (2)	C6—C7—C8—C9	−1.1 (6)
S1—Cd—N1—C1	178.8 (3)	C7—C8—C9—C4	0.8 (6)
S1^i^—Cd—N1—C1	23.7 (3)	C5—C4—C9—C8	−0.3 (5)
N1^i^—Cd—N1—N2	111.98 (19)	C3—C4—C9—C8	179.3 (3)
S1—Cd—N1—N2	−6.76 (17)	N1—N2—C10—S1	−2.2 (4)
S1^i^—Cd—N1—N2	−161.87 (15)	N1—N2—C10—S2	176.47 (19)
C1—N1—N2—C10	−177.9 (3)	Cd—S1—C10—N2	−3.1 (3)
Cd—N1—N2—C10	6.8 (3)	Cd—S1—C10—S2	178.22 (14)
N1^i^—Cd—S1—C10	−95.26 (13)	C11—S2—C10—N2	−10.3 (3)
N1—Cd—S1—C10	4.21 (13)	C11—S2—C10—S1	168.62 (18)
S1^i^—Cd—S1—C10	138.83 (11)	C10—S2—C11—C12	−169.8 (2)
N2—N1—C1—C2	176.4 (2)	S2—C11—C12—C13	86.4 (3)
Cd—N1—C1—C2	−9.0 (4)	S2—C11—C12—C17	−92.0 (3)
N1—C1—C2—C3	178.8 (3)	C17—C12—C13—C14	−0.6 (6)
C1—C2—C3—C4	176.1 (3)	C11—C12—C13—C14	−179.1 (3)
C2—C3—C4—C5	−3.2 (5)	C12—C13—C14—C15	0.7 (6)
C2—C3—C4—C9	177.2 (3)	C13—C14—C15—C16	0.2 (6)
C9—C4—C5—C6	0.1 (5)	C14—C15—C16—C17	−1.1 (6)
C3—C4—C5—C6	−179.5 (3)	C13—C12—C17—C16	−0.2 (5)
C4—C5—C6—C7	−0.3 (5)	C11—C12—C17—C16	178.2 (3)
C5—C6—C7—C8	0.8 (5)	C15—C16—C17—C12	1.1 (6)

Symmetry code: (i) −*x*+1, *y*, −*z*+3/2.

### Hydrogen-bond geometry (Å, º)

**Table:**

*D*—H···*A*	*D*—H	H···*A*	*D*···*A*	*D*—H···*A*
C13—H13···S2^ii^	0.95	2.75	3.690 (4)	170

Symmetry code: (ii) *x*, −*y*+1, *z*+1/2.
